# Modulation of colonic function in irritable bowel syndrome rats by electroacupuncture at ST25 and the neurobiological links between ST25 and the colon

**DOI:** 10.3389/fnins.2022.930489

**Published:** 2022-11-24

**Authors:** Lili Zhang, Cheng Yu, Biwei Chen, Yuqiao Chao, Haiyan Zhang, Qinyu Zhao, Kaiwei Yang, Yujiao Zhang, Shaozong Chen

**Affiliations:** ^1^College of Acupuncture and Massage, Shandong University of Traditional Chinese Medicine, Jinan, China; ^2^Department of Traditional Chinese Medicine, Affiliated Hospital of Shandong University of Traditional Chinese Medicine, Jinan, China; ^3^Institute of Acupuncture and Moxibustion, Shandong University of Traditional Chinese Medicine, Jinan, China

**Keywords:** irritable bowel syndrome (IBS), Tianshu (ST25), colon, neurophysiology, double fluorescent neural tracing technique

## Abstract

Irritable bowel syndrome (IBS) is a chronic functional gastrointestinal disease characterized by abdominal pain and defecation disorders. Acupuncture therapy positively affects IBS, with ST25 being the main point. However, ST25 has mostly been used in conjunction with other acupoints. This study aimed to observe the therapeutic effect of electroacupuncture at ST25 alone in IBS and the neurobiological mechanism of ST25 associated with the colon. First, we observed the effect of electroacupuncture at ST25 on the visceral pain threshold and slow-wave discharge of the colon in IBS model rats. Second, we explored the neurobiological mechanism of ST25 associated with the colon using a neural tracer technique. The results showed that (1) electroacupuncture at ST25 alone can alleviate visceral hypersensitivity and restore normal slow-wave frequency and rhythm of the colon in IBS rats; (2) there is a close neuroanatomical connection between ST25 and the colon, i.e., in the dorsal root ganglion (DRG), ST25 is similar in innervation to the colon, mainly in the T8–L1 segment, while the presence of double-labeled positive neurons is present in a part of the DRG; retrogradely labeled motor neurons associated with ST25 were observed in the anterior horn of the spinal cord, and retrogradely labeled sympathetic postganglionic neurons associated with ST25 were observed in the sympathetic nerve chain. These findings suggested that the DRGs and the dorsal horn of the spinal cord are important targets for electroacupuncture at ST25 to reduce visceral hypersensitivity in IBS rats. The sympathetic ganglia may be an important site for ST25 to regulate intestinal motility. The neurobiological mechanism of ST25 action in IBS rats should be further investigated in the future by combining related techniques, such as pseudorabies virus, optogenetics, calcium imaging, and electrophysiology.

## Introduction

Irritable bowel syndrome (IBS) is a common functional gastrointestinal disorder (Buono et al., 2017; Frandemark et al., 2018) characterized by abdominal pain accompanied by changes in stool characteristics and frequency (Sperber et al., 2017). Its complex pathogenesis remains unclear, but some studies have confirmed that IBS is closely related to visceral allergy and altered intestinal motility (Ford et al., 2020). The efficacy of acupuncture in treating IBS is confirmed by its ability to improve visceral hypersensitivity (Liu et al., 2015; Zhao et al., 2017) and adjust colonic motility (Manheimer et al., 2012; Yan et al., 2019; Pei et al., 2020; Nee and Lembo, 2021). ST25 is the main acupoint used in acupuncture to treat IBS (Chen and Chen, 2021), but ST25 has mostly been studied in conjunction with other acupoints, with the use of ST25 alone being rare (Ma et al., 2009; Zhu et al., 2017; Chen et al., 2019). Furthermore, no study has confirmed the mechanism of association between ST25 and the colon from a neuroanatomical perspective.

In this study, we used neuroelectrophysiological techniques to observe the effects of electroacupuncture (EA) at ST25 alone on visceral sensitivity and colonic motility in IBS rats. Then a double fluorescent neural tracing technique with Alexa Fluor 488 and 594 conjugates of cholera toxin subunit B (AF488/594-CTB) was used for figuring out the neuronal correlation between ST25 and the colon. Tao et al. (1981) was a pioneer in applying nerve tracing techniques to acupuncture, and retrograde nerve tracing has been successfully used to study the neuroanatomical connections of acupoints–target organs (Cui et al., 2015, 2019; Xu et al., 2021; Zhang et al., 2021). By injecting different nerve tracers, sensory, sympathetic, and motor neurons associated with ST25 and the colon can be observed. The present study could reveal the neurophysiological mechanism of ST25 effects on IBS at the cellular level.

## Materials and methods

### Animals

We enrolled a total number of 71 male Sprague–Dawley (SD) rats (200 ± 20 g). Rats were purchased from Beijing Weitonglihua Experimental Animal Technology Co., Ltd. (Beijing, China) [experimental animal license number: SYXK (LU) 20170022]. Animal experiments were performed in accordance with the Guide for the Care and Use of Laboratory Animals (published by the United States National Institutes of Health) and approved by the Institutional Animal Care and Research Advisory Committee of the Shandong University of Traditional Chinese Medicine. The study protocol was approved by the ethics committee of the Shandong University of Traditional Chinese Medicine Experimental Animal Welfare Ethics Review Committee (reference number: SDUTCM20200620001). Rats were housed in a clean-grade laboratory (22 ± 2°C, 12-h/12-h light/dark cycle), fed and watered freely, with air delivery 6–10 times/h and 50–70% humidity. Before the experiment, rats need to be adaptively raised in the standard experimental environment for 7 days.

### Experimental design

The experiment was divided into two parts. First, the abdominal withdrawal reflex (AWR) test, extra-abdominal oblique electromyography (EMG) recording, and colonic EMG were used to determine whether electroacupuncture at ST25 could alleviate visceral hypersensitivity and restore colonic motility in IBS rats. Second, the neurobiological mechanism of ST25 associated with the colon was observed in the DRG, sympathetic ganglia, and spinal cord using a nerve tracing technique.

**FIGURE 1:**
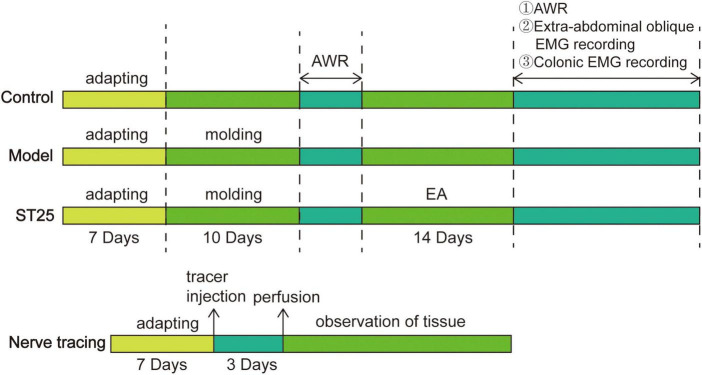
A flowchart of experimental protocol: intervention timeline for the control group, model group, ST25 group, and nerve tracing group. AWR, Abdominal withdrawal reaction.

In the first part of the study, 21 rats were randomly assigned to the control group, and the remaining rats were established as IBS rat models based on water avoidance stress (WAS). After modeling, the AWR test was performed uniformly, and the IBS rats were subsequently randomly divided into the model and ST25 groups, with 21 rats in each group. The ST25 group received EA for a total of 14 days. To eliminate the effect of grabbing and fixing, the rats in the control and model groups were grabbed and fixed every day without EA. After treatment completion, eight rats were randomly selected from the control, model, and ST25 groups for the AWR test; five for abdominal oblique EMG; and eight for colonic EMG. In the second part of the study, eight healthy SD rats were selected to explore the neuroanatomical relationship between ST25 and the colon. After 72 h of nerve tracer injection, the rats were perfused and sampled. Seventy-two hours after tracer injection, the rats were perfused for sampling. The experimental protocol is shown in Figure 1.

### Modulatory effect of electroacupuncture at ST25 on colonic function in irritable bowel syndrome model rats

#### Establishment of the irritable bowel syndrome rat model

The IBS rat model was established using the WAS method (Bradesi et al., 2005). A block (10 cm × 8 cm × 8 cm) was placed in the middle of a transparent tank (45 cm × 25 cm × 25 cm), and water (25°C ± 2°C) was injected into the tank to ensure that the water surface was 1 cm below the top of the block. Rats were kept on the block for 1 h each day from 9:00 to 10:00 A.M. for 10 days consecutively.

#### Electroacupuncture treatment

The positioning of ST25 was referred to as “Experimental Acupuncture” (Yu et al., 2021), and combined with the positioning of human acupuncture points, ST25 was located approximately 5-mm paracentral to the upper 7/12 and lower 5/12 of the line connecting the raphe and the pubic symphysis in rats. Rats in the EA group were restrained in a special frame for electroacupuncture. For EA at ST25, two pairs of needles (diameter, 0.3 mm; length, 15 mm; Huatuo, Suzhou, China) were bilaterally inserted 3-mm deep into the abdominal muscle layers. The needles were then connected to an EA therapeutic stimulator (Nanjing Jisheng Medical Science and Technology, Ltd., Nanjing, China). The parameters of the stimulator were applied for a sparse wave at 2/15 Hz, with a current intensity of 1 mA. EA was administered for 20 min daily for 14 days (Xu et al., 2018; Song et al., 2020).

#### Abdominal withdrawal reflex scores

Eight rats were selected for the AWR score. Based on previous studies (Al-Chaer et al., 2000; Yu et al., 2012; Tao et al., 2021), the sensitivity of the colon to colorectal distention (CRD) was evaluated by calculating the AWR score. Before the experiment, rats were given free access to water but were food-deprived for 8–12 h. Rats were slightly anesthetized with 1% isoflurane (RWD Life Science Co., Ltd., Shenzhen, China) in an induction box. The distension balloon was emptied and coated with paraffin oil and then inserted into the anus of the rats at a depth of 6 cm, and the balloon was gently bound to the tail of the rats to prevent it from slipping out. The rats were placed on a platform and restrained in a transparent plastic box (20 cm × 8 cm × 8 cm) and allowed to acclimatize for 15–20 min before the AWR test. There were four pressure levels: 20, 40, 60, and 80 mmHg. Each CRD lasted approximately 20 s and was repeated three times. AWR scores were generated blindly, without subjective judgment, and the average scores were calculated. The specific rules of AWR scoring were as follows: 0 denotes no behavioral response to CRD; 1 denotes that at the beginning of stimulation, head movement occasionally occurs; 2 denotes mild contraction of abdominal muscles, no lifting; 3 denotes the abdominal muscles contracted strongly, and the abdominal rather than pelvic structures were lifted from the platform; and 4 denotes the body arched and the pelvic structure lifted from the platform. The pain threshold was defined by a stimulus intensity that evoked a visible contraction of the abdominal wall when the AWR score was 3 (Su et al., 2020).

#### Extra-abdominal oblique electromyography recording

Five rats were selected for recording the EMG recording the abdominal oblique muscle, and the rats were anesthetized with 2% isoflurane for deep anesthesia. The temperature of the rats was maintained at approximately 37°C using a temperature control system for the experimental animals. Under an aseptic surgical operation, a longitudinal incision was made along the midline of the abdomen and the midpoint of the anterior superior iliac spine. The skin and subcutaneous fat were incised to expose the abdominal oblique muscle: electrodes were then implanted into the muscular layer. After the electrodes were implanted, a glycerol-lubricated balloon was slowly inserted 6–7 cm through the anus of the rats and fixed. The balloon was connected to a pressure sensor to enable immediate pressure detection. Thereafter, rats were slightly anesthetized with 1% isoflurane and stabilized for 15 min before CRD. The colon was dilated with a pressure of 20, 40, 60, and 80 mmHg, lasting 20 s, and the interval between stimuli was 2 min. EMG signals were input into the computer through amplifiers and AD signal converters. PowerLab data acquisition and analysis systems (AD Instruments, Lab Chart 7.0, NSW, Australia) were used to record the discharge activities of the external oblique muscles of rats under different expansion pressures. EMG analysis was performed by measuring the area under the curve (AUC) of the EMG signal in response to CRD stimulations at different pressure. The analysis period was 20 s. The net value of each CRD was calculated by subtracting the AUC of the baseline (20 s) before each CRD (Zhu et al., 2018).

#### Colonic electromyography recording

Eight rats were selected for colonic intestinal electrical recording, and two 20-cm insulated platinum wire electrodes were prepared, with the insulating film removed at both ends 1 cm from the end to expose a 0.5-cm platinum wire for use as the electrodes. Rats were anesthetized with 2% isoflurane for deep anesthesia, and a pair of electrodes were embedded in the colon at a distance of approximately 2 cm from the end of the cecum, with the electrodes approximately 3-mm apart and the connection line perpendicular to the long axis of the cavity organ. Colonic intestinal electrical signals were entered into the computer through the amplifier and AD signal converter. After the colonic discharge was stabilized, the PowerLab data acquisition and analysis system was used to record for 1 h. Select a stable signal for 1 min as a period, each group selected five time periods for analysis (Cui et al., 2022).

### Mechanism of association between ST25 and the colon in neuroanatomy

#### Surgical procedures and tracer injection

Under 2% isoflurane respiratory anesthesia, 4 μl of 0.1% AF594-CTB solution (Invitrogen-Molecular Probes, Eugene, OR, USA) was injected subcutaneously and muscularly into the right side of ST25 using a Hamilton micro-syringe (Hamilton Company, Reno, NV, United States) (Zhang et al., 2021). The positioning of ST25 was referred to as “Experimental Acupuncture” (Yu et al., 2021), and combined with the positioning of human acupuncture points, ST25 was located at approximately 5-mm paracentral to the upper 7/12 and lower 5/12 of the line connecting the raphe and the pubic symphysis in rats. The abdomen was opened under surgical asepsis to expose the colon. The operation was performed under a body microscope, and 10 μl of 0.1% AF488-CTB solution (Invitrogen-Molecular Probes, Eugene, OR, USA) was slowly injected into the proximal colon (ascending colon and 1/2 transverse colon) at multiple points in the muscle layer, and the needle was left for 5 min after the injection to prevent extravasation of the tracer from affecting the experimental results. The rats were placed back into a cage in the feeding box after they were fully awake.

#### Perfusion

Seventy-two hours after tracer injection (Wang et al., 2019; Zhang et al., 2021), the rats were perfused for sampling, anesthetized intraperitoneally with 10% urethane (1 ml/100 g), and fixed by perfusion with 0.9% sodium chloride solution and 4% paraformaldehyde through the heart. Subsequently, the spinal ganglia of the T1–L6 segments, spinal cord, sympathetic chain, local tissues of ST25, and colon were dissected out, post-fixed for 2–3 h, and replaced into 0.1 mol/L PB (pH 7.4) containing 25% sucrose for dehydration. Subsequently, they were placed in a 4°C refrigerator to wait for the tissue to sink naturally.

#### Preparation and observation of tissue sections

The DRGs, spinal cord, and sympathetic ganglia were cut at a thickness of 25 μm using a freezing microtome (Leica HM430, Wetzlar, Germany), and all consecutive sections were collected. Local ST25 tissue was cut into 20-μm thick sections (Wang et al., 2018; Zhang et al., 2021). All sections were mounted on slides, sealed with a drop of an anti-fluorescence quenching sealer (Abcam, Cambridge, MA, USA), and observed under laser confocal (ZEISS LSM880 + Fast Airyscan, Germany) and inverted fluorescence microscope (ZEISS Axio Scope AI, Germany) microscopes.

### Statistical analysis

Data are expressed as mean ± standard error. Differences among multiple groups were evaluated by the analysis of variance (one-way or two-way ANOVA), and *p* < 0.05 was set to indicate statistical significance. GraphPad Prism 8.0.2 (La Jolla, CA, United States) and Adobe Illustrator 2020 (Adobe, San Jose, CA, USA) were used for the analysis and graphing.

## Results

### Modulatory effect of electroacupuncture at ST25 on colonic function in irritable bowel syndrome model rats

#### Evaluation of the animal model

**FIGURE 2:**
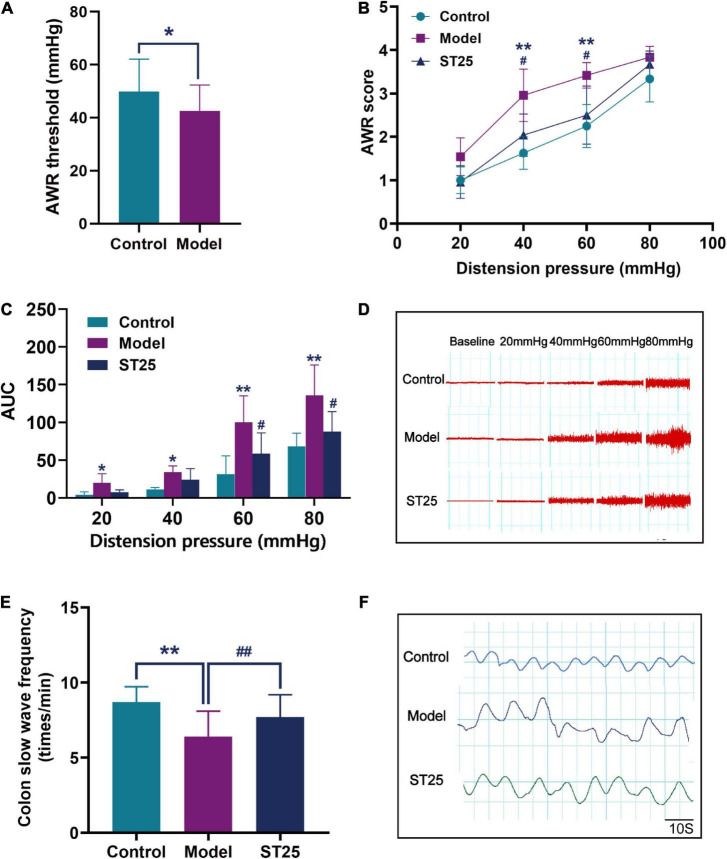
The effect of electroacupuncture at ST25 on pain threshold and colonic motility in irritable bowel syndrome (IBS) rats. **(A)** Validation of the animal model of the IBS model. The visceral pain threshold pressure was measured using the abdominal withdrawal reflex (AWR) test in IBS (model) rats (*n* = 42) and control (normal) rats (*n* = 21). **(B)** AWR scores under different pressure (20, 40, 60, and 80 mmHg), *n* = 8. **(C)** The area under the curve (AUC) of EMG activity in the external oblique muscle in response to graded distension pressures, *n* = 5. **(D)** Representative EMG under different pressure (20, 40, 60, and 80 mmHg). **(E)** Colon slow-wave frequency, *n* = 8. **(F)** Representative colonic EMG graphs. Data are expressed as mean ± standard error, *p < 0.05, **p < 0.01 compared with the control group. ^#^*p* < 0.05, ^##^*p* < 0.01 compared with the model group.

Following WAS, we observed the AWR scores of the rats by CRD. Results demonstrated that the visceral pain threshold of rats in the model group was significantly lower than that of controls (*p* < 0.05; (Figure 2A). Thus, IBS was confirmed to have been induced successfully in model rats.

#### Effect of electroacupuncture at ST25 on visceral hypersensitivity in irritable bowel syndrome rats

First, the AWR test was used to evaluate the analgesic effect of electroacupuncture at ST25 on visceral hyperalgesia (Figure 2B). Under the stimulations at 40 and 60 mmHg, the AWR scores were significantly higher in the IBS model rats than in the control group (*p* < 0.01). Under pressure stimulations at 40 and 60 mmHg, the AWR scores of rats were significantly lower in the ST25 group than in the model group (*p* < 0.05). In addition, the analgesic effect of electroacupuncture at ST25 on visceral nociceptive hyperalgesia was assessed by EMG recording of the external oblique abdominal muscles in the dilated colorectal state (Figures 2C,D). The EMG recordings showed results consistent with those of the AWR test. The AUC of EMG in the model group was significantly higher compared with that in the control group (20 mmHg, *p* < 0.05; 40 mmHg, *p* < 0.05; 60 mmHg, *p* < 0.01; 80 mmHg, *p* < 0.01). EMG was lower in the ST25 group than in the model group (60 mmHg, *p* < 0.05; 80 mmHg, *p* < 0.05).

#### Effect of electroacupuncture at ST25 on colonic motility in irritable bowel syndrome rats

The effect of electroacupuncture at ST25 on pain threshold and colonic motility in IBS rats is shown in Table 1 and Figures 2E,F. Compared with the control group, the colonic slow-wave frequency of the model rats was significantly lower (*p* < 0.01), while the colonic slow-wave rhythm was disturbed. Compared with the model group, the frequency of colon slow-wave rhythm was significantly increased in the ST25 group (*p* < 0.01), while the colon slow-wave morphology gradually returned to normal after electroacupuncture at ST25.

**TABLE 1:** Effect of electroacupuncture at ST25 on colonic slow-wave discharge in irritable bowel syndrome (IBS) model rats.

Groups	*n*	Colonic slow-wave frequency (times/min)
Control group	8	8.70 ± 1.02
Model group	8	6.40 ± 1.69**
ST25 group	8	7.70 ± 1.49^##^

Data are represented as mean ± SEM. **p < 0.01 compared with the control group, ^##^*p* < 0.01 compared with the model group.

### Mechanism of association between ST25 and the colon in neuroanatomy

#### Distribution of neural tracers in the colon and ST25

**FIGURE 3:**
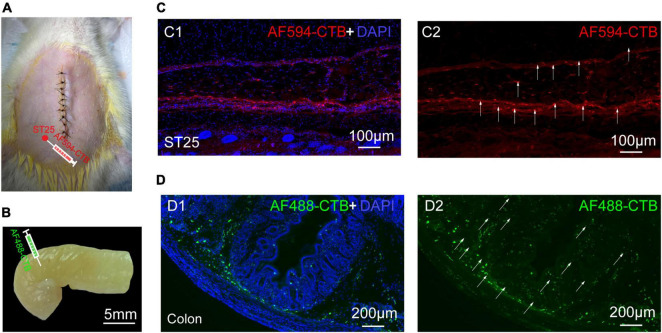
The site of nerve tracer injection and distribution of proximal colonic and ST25 nerve tracers **(A)** The ST25 injection site. **(B)** The colon injection site. **(C)** Representative section of the ST25. **(D)** Representative section of the colon.

The proximal colon and ST25 tissues were sectioned for observation. The section of the proximal colon showed that the neural tracer was widely dispersed in the muscular and submucosal layers of the colon. The neural tracer in ST25 was also widely distributed, mainly around the thoracic nerves (Figures 3A,B). Figures 3C,D are tissue sections of ST25 and the colon.

#### Positive labeling of the dorsal root ganglion

**FIGURE 4:**
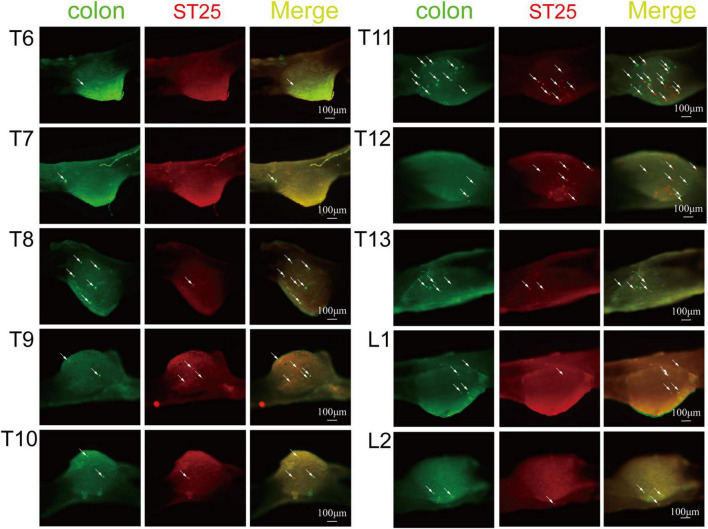
The positive labeling of dorsal root ganglia (DRGs) (not sectioned) under fluorescence microscopy. Representative photomicrographs showing the distribution of AF488/594-CTB-labeled sensory neurons in T6–L2 DRGs.

**FIGURE 5:**
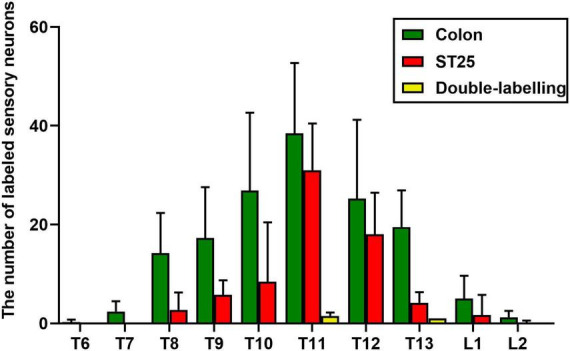
The number of labeled sensory neurons in the thoracic (T) and lumbar (L) dorsal root ganglia (DRGs) (mean ± standard error, *n* = 8).

**FIGURE 6:**
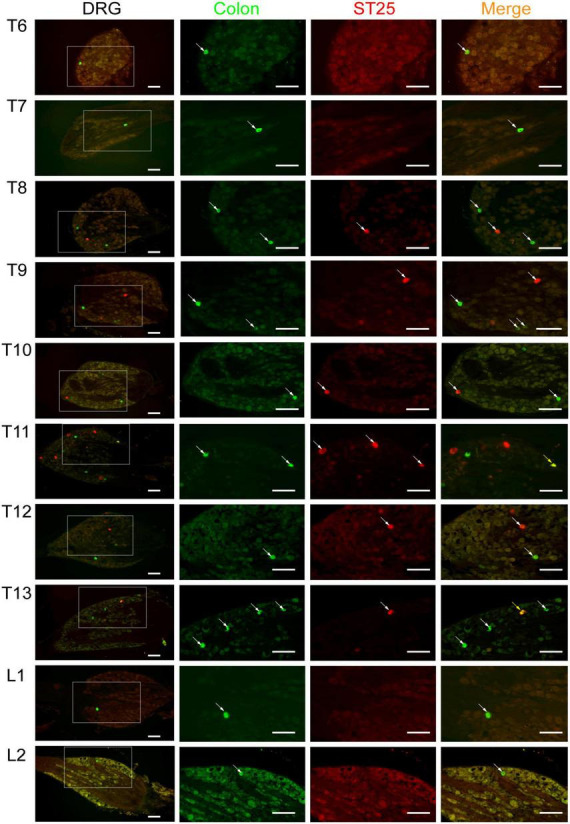
The neural tracing of the colon and ST25 in the dorsal root ganglia (DRGs) (sectioned). Representative and magnified photomicrographs showing the distribution of AF488/594-CTB-labeled sensory neurons in the T6–L2 DRGs. The double-labeled neurons with AF488/594-CTB presenting in yellow. Scale bars = 100 μm.

AF594-CTB excitation fluorescence was red, while AF488-CTB excitation fluorescence was green. The labeled sensory neurons corresponded to ST25 and the proximal colon, respectively. First, the dehydrated DRGs were directly observed for positive labeling under a fluorescence microscope (Figure 4). The results showed that the positive labeling of the proximal colon was distributed in the thoracic (T) T6 to lumbar (L) 2 DRGs and the positive labeling of ST25 was distributed in the T8–L2 DRGs, both of which were concentrated in the T8–T13 DRGs. Second, the positively labeled neurons in all sections of DRGs were counted (Figures 5, 6). The positive sensory neurons labeled by ST25 in order of number were as follows: T11 (31 ± 9.47) > T12 (18 ± 8.45) > T10 (8.43 ± 12.04) > T9 (5.75 ± 2.99) > T13 (4.17 ± 2.14) > T8 (2.75 ± 3.5) > L1 (1.67 ± 4.08) > L2 (0.17 ± 0.41). The number of positive sensory neurons labeled in the proximal colon was in the following order: T11 (38.44 ± 14.25) > T10 (26.88 ± 15.75) > T12 (25.25 ± 15.95) > T13 (19.5 ± 7.41) > T9 (17.29 ± 10.29) > T8 (14.25 ± 8.12) > L1 (5.00 ± 4.65) > T7 (2.4 ± 2.07) > L2 (1.25 ± 1.28) > T6 (0.25 ± 0.50). A few double-labeled neurons of ST25 and the colon were found in the T11 and T13 DRGs.

#### Positive labeling of the spinal cord

**FIGURE 7:**
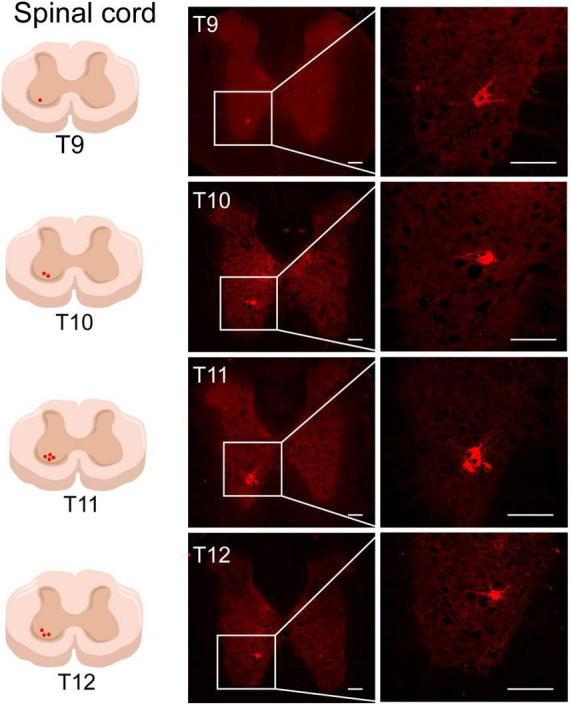
The neural tracing of ST25 in the spinal cord. Representative and magnified photomicrograph showing the distribution of AF594-CTB-labeled motor neurons in the T9–T12 spinal cord. Scale bars = 100 μm.

Sections of the thoracic spinal cord were observed, and AF594-CTB-labeled motor neurons were observed in all T9–T12 spinal cord, mainly in the anterior horn of the spinal cord (Figure 7), associated with local motor innervation of ST25. No AF488-CTB-labeled motor neurons were observed. No positive sympathetic preganglionic neurons were observed in the lateral horn of the spinal cord, suggesting that sympathetic preganglionic neurons cannot directly innervate ST25 or the proximal colon.

#### Positive labeling of the sympathetic chain

**FIGURE 8:**
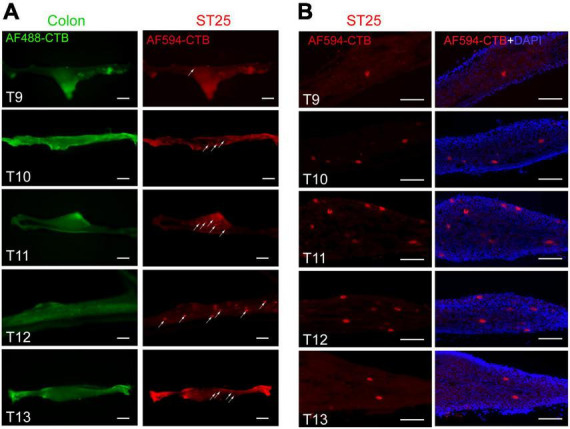
The positive labeling of the sympathetic chain. **(A)** Positive labeling of sympathetic chain (not sectioned) under fluorescence microscopy. **(B)** Representative photomicrographs showing the distribution of AF594-CTB-labeled post-sympathetic neurons in the sympathetic chain (sectioned) at the thoracic segments. Scale bars = 100 μm.

The thoracic segment sympathetic chain was observed directly under fluorescence microscopy, and positive neurons labeled with AF594-CTB were observed in the T9–T13 segments (Figure 8A), and the most positive labeling was observed in T10–T12 segments. No sympathetic neurons labeled with AF488-CTB were observed. Similar results were observed in the thoracic segment sympathetic chain sections (Figure 8B).

## Discussion

Typical pathological features of IBS include visceral hypersensitivity and altered colonic motility (Buono et al., 2017; Frandemark et al., 2018). Visceral hypersensitivity refers to the phenomenon in which the threshold for causing pain or discomfort to the viscera is lowered, and the viscera becomes uncomfortable with physiological stimuli or reacts strongly to injurious stimuli (Zhou and Verne, 2011). Visceral hypersensitivity is closely related to peripheral and central sensitizations (Al-Chaer and Traub, 2002). The number of nerve fiber endings in the colonic mucosa of patients with IBS is significantly increased (Yu et al., 2012), and abnormal cytokine expression and an increase in mast cells and intestinal chromophores stimulate intestinal afferent nerve endings leading to peripheral sensitization (Camilleri et al., 2017). Second, pentraxin, substance P, and calcitonin gene-related peptide can directly activate nerve endings or promote the release of pain mediators (Yu et al., 2012; Camilleri et al., 2017; Xu et al., 2017), contributing to peripheral sensitization, which subsequently contributes to central sensitization (Ji et al., 2018).

The dorsal root ganglion is an important structure for sensory transmission and regulation, including pain (Hogan, 2010). The sensitization of DRG neurons is an important factor in the induction of chronic pain (Ji et al., 2014). Patch-clamp recordings show that the excitability of voltage-gated K (+) channels in DRG neurons of IBS rats dominating the colon increases (Luo et al., 2011). The neurotransmitters and mediators released by primary neurons continue to activate the spinal dorsal horn-related receptors (Basso and Altier, 2017; Inoue, 2019) and increase their expression (Zhou et al., 2019; Wang et al., 2021). Abnormal excitation of spinal dorsal horn neurons promotes central sensitization. Therefore, the body will produce pain after physiological or mild stimulation, and peripheral sensitization is an important factor leading to central sensitization. The DRG and spinal cord also play important roles in visceral pain. In the relationship between acupoints and target organs, the DRG and spinal cord are also key components of body signal and visceral signal integration. Some scholars (Chen, 2019) have summarized the neuroanatomical relationship between acupoints and the target organs into two aspects: the spinal cord and peripheral mechanisms (DRG mechanism). The spinal cord mechanism includes the spinal cord reflex, dorsal root reflex, and the common convergence mechanism. Peripheral mechanisms include short axon reflexes and long axon reflexes; therefore, the DRG and spinal cord are important parts of the nerve connection between acupoints and target organs.

Dorsal root ganglion is a new target of neuromodulation (Esposito et al., 2019), and its stimulation can treat chronic intractable neuropathic pain (Liem et al., 2015). Acupuncture at ST25 downregulates the expressions of GFAP and P2 × 3 receptors in the DRG and spinal cord, BDNF and TrkB proteins and mRNA in the colon and DRG, and TRP phosphatidylinositol (Prit) in DRG and capsaicin receptor (TRPV1) in the colonic mucosa (Weng et al., 2015; Ji and Huang, 2020), thus improving colonic pain sensitivity in IBS model rats (Chen et al., 2021; Jin et al., 2021). Thus, the DRG and the dorsal horn of the spinal cord may be key targets of ST25 in relieving visceral pain in rats with IBS.

Based on the aforementioned theory, this study observed the modulating effect on visceral hypersensitivity by electroacupuncture of ST25 alone, followed by we used retrograde neural tracers AF488-CTB and AF594-CTB (Lanciego and Wouterlood, 2020) to observe the neuroanatomical connections between the colon and ST25. The results showed that IBS rats had increased visceral sensitivity and decreased pain threshold, and EA at ST25 could relieve visceral pain in IBS rats. Even though most of the neurons were separately labeled with AF488-CTB or AF594-CTB, they locate adjacently in the DRGs at the same spinal segments, and some of the sensory neurons were simultaneously labeled with both AF488/594-CTB. Retrograde-labeled sensory neurons of ST25 were mainly distributed in the T8–L1 DRGs (concentrated in the T11 segment), and the sensory neurons of the colonic were mainly distributed in the T6–L2 DRGs (concentrated in the T11 segment). There were some co-labeled positive neurons in the T11 and T13 DRGs. Sinclair et al. (1948) proposed the branching projection theory of entrapment pain: There might be multiple branches of the DRGs cell perineurium that innervate the visceral organs and somatic structures. In 1979, the existence of DRG axon bifurcation was confirmed by fluorescein double labeling (Taylor and Pierau, 1982). The present study further confirms this theory. Thus, ST25 is closely associated with the colon in the primary sensory afferents, which may be an important neuroanatomical pathway for electroacupuncture at ST25 to modulate proximal colon function. However, in-depth studies are lacking on how sensory signals from the colon interact with stimulation signals from ST25 in DRG. In addition, at the spinal cord level, since this study used retrograde non-trans-synaptic nerve tracers, neurons in the dorsal and lateral horns of the spinal cord could not be observed. Only motor neurons innervating ST25 in the anterior horn of the spinal cord were seen, and the labeled motor neurons were only related to the innervation of local skeletal muscles of ST25, indicating that acupuncture at ST25 does not affect the colon through motor conduction pathways. The local anatomy of ST25 shows that the ventral rectus muscle distributed under the acupuncture area is mainly innervated by motor neurons in the T7–L1 segments (Yang et al., 2009; Grinsell et al., 2020), which is consistent with the nerve tracing results.

Another typical feature of IBS is altered colonic motility manifested by abnormal colonic electromyographic activity, repeated contractions of the small intestine and colon with abdominal pain (Snape et al., 1977; Sullivan et al., 1978), and altered gastrointestinal or colonic transport (Kellow and Phillips, 1987; Spiller et al., 1987). The electrical activity of the colonic muscle is an objective electrophysiological indicator of colonic motility (Shafik et al., 2004). It manifests in two main types: a basic electrical rhythm, also known as slow-wave or electrical control activity, and an action potential, also known as fast wave or peak potential. The slow wave is a local potential, which originates from the longitudinal muscle and is a relatively regular periodic electrical activity that controls the contraction rhythm of the intestine and is always present whether contracted or not. The action potential is generated on top of the slow wave, which is consistent with the smooth muscle contraction and is the main initiator of the propulsive contraction (Ahmed and Ali, 2000; Shafik et al., 2001). The frequency and amplitude of colonic slow waves can reflect colonic function.

Based on the aforementioned theory, this study observed the regulating effect of electroacupuncture ST25 alone on the colonic slow wave of IBS rats. The experimental results showed that the colonic slow-wave frequency decreased and the slow-wave rhythm was irregular in IBS rats, suggesting that the colonic motility of IBS rats was abnormal. After electroacupuncture at ST25 treatment, the colonic electromyographic colonic slow-wave frequency and rhythm of IBS rats gradually tended to a normal level. Thus, ST25 has a good regulating effect on the colonic motility of IBS rats. The colon receives innervation from the central nervous system, the autonomic nervous system, and the endogenous enteric nervous system (Qin et al., 2007; Matsumura et al., 2010; Furness et al., 2014). The regulation of gastrointestinal motor function by acupuncture also depends mainly on the following three levels: the local enteric nervous system, the autonomic nervous system, and the central nervous system (Yu, 2020). The sympathetic nerve has an inhibitory effect on the gastrointestinal tract, and the parasympathetic nerve has an excitatory effect on the gastrointestinal tract. Some studies (Wang et al., 1992; Zhu et al., 1996) have performed retrograde nerve tracing on the colon and revealed the presence of retrogradely labeled positive neurons in both the anterior vertebral ganglia and the sympathetic trunk ganglion of the thoracic segment. The somatic vascular smooth muscle receives sympathetic innervation, and the nerve fibers localized to the acupuncture point originate from the somatic nerve as well as the vascular plexus (Zhang et al., 2021). Studies (Qin et al., 2013a,b; Li et al., 2021) have confirmed that ST25 can regulate gastrointestinal motility by activating somatosensory or parasympathetic pathways; therefore, the sympathetic trunk ganglion in the peripheral nervous system may be a target of ST25 in regulating colonic function. Based on the aforementioned theory, this study also observed the sympathetic neurons of the colon and ST25 by nerve tracer technique. The results indicated that there were sympathetic postganglionic neurons retrogradely labeled by ST25 in the sympathetic chain, indicating that the nerve fibers of ST25 come from the somatic nerve and the peripheral vascular plexus in the acupuncture area. Thus, acupuncture at ST25 has a modulating effect on sympathetic nerves. However, this study used a retrograde non-trans-synaptic nerve tracer and failed to show positive neurons of the colon in the sympathetic chain. Further experiments should be conducted using trans-synaptic nerve tracers for further investigation. Previous studies (Li et al., 2007) have shown that abdominal acupoints can increase sympathetic discharge to inhibit gastric motility; however, the direction of regulation of intestinal motility is closely related to the different segments of the intestine and the disease state of the body. Acupuncture at ST25 could activate the somato-parasympathetic reflex to promote the motility of the colon in constipated rats (Qin et al., 2013a).

Therefore, the regulatory effect of electroacupuncture at ST25 on colonic motility in IBS rats may be the result of a balance between the somato-sympathetic reflex and somato-parasympathetic reflexes. Subsequently, the mechanism of ST25 and the colon in the parasympathetic reflex should be studied using a trans-synaptic nerve tracer.

## Conclusion

**FIGURE 9:**
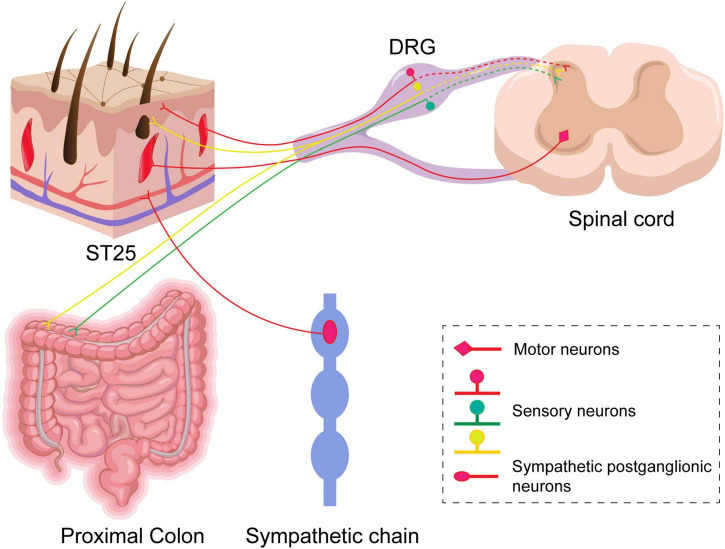
A schematic diagram of sensory and motor innervations of ST25 and the colon in rats. Labeled sensory neurons in the dorsal root ganglia (DRGs) innervate ST25 and the colon, respectively, and their central synapses project to the posterior horn of the spinal cord. There are a small number of co-labeled neurons in the DRGs. Postganglionic neurons in the sympathetic chain and motor neurons in the anterior horn of the spinal cord are relative to ST25 only.

The typical manifestations of IBS are visceral hypersensitivity and altered colonic kinetics, and acupuncture is effective in treating IBS. However, studies are lacking on the effects of acupuncture at ST25 alone on visceral pain and colonic kinetics in IBS or the mechanism of the association between ST25 and the colon in neuroanatomy. First, we confirmed that acupuncture at ST25 alone could alleviate visceral hypersensitivity and regulate colonic motility in IBS rats. Second, we used neural tracing technology to study the neuroanatomical relationship between ST25 and the colon and to explain the therapeutic mechanism of ST25 in IBS rats. The present study confirmed that ST25 and the colon are closely associated with neuroanatomy (Figure 9). There is a direct sensory correlation between ST25 and the colon, and DRG is a key target for electroacupuncture at ST25 to alleviate visceral pain in IBS rats. There may be a close relationship between ST25 and the colon in sympathetic postganglionic neurons, which may be another important target for ST25 to regulate colon function, and this is an important question to be solved in future studies. In our study, we used retrograde non-trans-synaptic neural tracers that primarily tracked first-order neurons associated with the colon and ST25, and therefore failed to observe the neural circuits in the dorsal and lateral horns of the spinal cord and the brain. In the follow-up study, we aim to investigate the neurobiological mechanism of acupuncture at ST25 for IBS combined with pseudorabies virus, optogenetic, calcium imaging, and patch clamp.

## Data availability statement

The raw data supporting the conclusions of this article will be made available by the authors, without undue reservation.

## Ethics statement

The animal study was reviewed and approved by the Ethics Committee of Shandong University of Traditional Chinese Medicine Experimental Animal Welfare Ethics Review Committee. Written informed consent was obtained from the owners for the participation of their animals in this study.

## Author contributions

SC designed the experiment. LZ, CY, BC, YC, HZ, QZ, KY, and YZ conducted the experiments. LZ, CY, and BC analyzed the data. LZ, YZ, and CY wrote the manuscript. All authors read and approved the final version of the manuscript accepted for publication.
