# Corrigendum: Modulation of colonic function in irritable bowel syndrome rats by electroacupuncture at ST25 and the neurobiological links between ST25 and the colon

**DOI:** 10.3389/fnins.2023.1177384

**Published:** 2023-03-16

**Authors:** Lili Zhang, Cheng Yu, Biwei Chen, Yuqiao Chao, Haiyan Zhang, Qinyu Zhao, Kaiwei Yang, Yujiao Zhang, Shaozong Chen

**Affiliations:** ^1^College of Acupuncture and Massage, Shandong University of Traditional Chinese Medicine, Jinan, China; ^2^Department of Traditional Chinese Medicine, Affiliated Hospital of Shandong University of Traditional Chinese Medicine, Jinan, China; ^3^Institute of Acupuncture and Moxibustion, Shandong University of Traditional Chinese Medicine, Jinan, China

**Keywords:** irritable bowel syndrome (IBS), Tianshu (ST25), colon, neurophysiology, double fluorescent neural tracing technique

In the original article, there was an error in the Funding statement. [The funding statement for “This study was supported by the project of the National Key R&D Program of China (No. 2022YFC3500602), the project of the National Key R&D Program of China (No. 2019YFC1712105), and the Major Basic Research Project of Shandong Natural Science Foundation (No. ZR2020ZD17)”]. The correct **Funding** statement appears below:

“This study was supported by the project of the National Key R&D Program of China (No. 2019YFC1712105), and the Major Basic Research Project of Shandong Natural Science Foundation (No. ZR2020ZD17).”

The authors apologize for this error and state that this does not change the scientific conclusions of the article in any way. The original article has been updated.

